# Monteagle as a Summer Resort

**Published:** 1884-09-20

**Authors:** 


### MONTEAGLE AS A SUMMER RESORT.

The managing editor of this journal, under an invitation from the Executive Committee of the Monteagle Assembly, visited the above beautiful and attractive place in the capacity of a lecturer. His address was delivered on the 20th of August to a large and intelligent audience; subject, "Science; its Progress, its Beauties as a Study and its Relations to Religion." On the latter branch of his theme he discussed the subject of Evolution. The address attracted marked attention and was most favorably commented upon by the Nashville press.

The Monteagle Assembly grounds are on the top of the Cumberland mountain, two thousand, two hundred feet above the level of the sea. The climate is cool and invigorating, and the attractions of the place many and of a rare order.

The plan of the organization is similar to that of the Northern Chattauqua, the scheme being the establishment of a magnificent summer resort, with moral, devotional and intellectual exercises as methods of diversion and entertainment. The locality is, in all respects, admirably adapted to the purpose. The enterprise is a noble one, and well worthy of the sympathy and encouragement of all good people.

